# Renal Denervation in Asia: 2025 Asia Renal Denervation Consortium (ARDeC) Consensus Statement Endorsed by the Hypertension Cardiovascular Outcome Prevention and Evidence in Asia (HOPE Asia) Network

**DOI:** 10.1161/HYPERTENSIONAHA.125.25333

**Published:** 2025-12-02

**Authors:** Kazuomi Kario, Amir Aziz Alkatiri, Jiro Aoki, Wan Azman Wan Ahmad, Yook-Chin Chia, Jose Nicolas M. Cruz, Ying-Hsiang Lee, Soo Teik Lim, Chengzhi Lu, Quang Ngoc Nguyen, Tiong Kiam Ong, Gurpreet S. Wander, Ji-Guang Wang, Yiu-Tung Anthony Wong, Nattawut Wongpraparut, Tzung-Dau Wang

**Affiliations:** 1 Department of Cardiovascular Medicine, Jichi Medical University School of Medicine, Shimotsuke, Japan (K.K.).; 2 Faculty of Medicine, Department of Cardiology and Vascular Medicine, Universitas Indonesia, National Cardiovascular Center Harapan Kita, Jakarta (A.A.A.).; 3 Department of Cardiovascular Medicine, St. Luke’s International Hospital, Tokyo, Japan (J.A.).; 4 Division of Cardiology (W.A.W.A.), University of Malaya, Kuala Lumpur, Malaysia.; 5 Department of Primary Care Medicine (Y.-C.C.), University of Malaya, Kuala Lumpur, Malaysia.; 6 Faculty of Medical and Life Sciences, Department of Clinical Medicine and Surgery, Sir Jeffrey Cheah Sunway Medical School, Sunway University, Selangor, Malaysia (Y.-C.C.).; 7 St. Luke’s Medical Center, Taguig City, Philippines (J.N.M.C.).; 8 Cardiovascular Medicine, MacKay Memorial Hospital and MacKay Junior College of Medicine, Nursing, and Management, Taipei City, Taiwan (Y.-H.L.).; 9 Department of Cardiology, Tianjin First Central Hospital, China (S.T.L.).; 10 Department of Cardiology of the Second Affiliated Hospital, Zhejiang University School of Medicine, Hangzhou, China (C.L.).; 11 Department of Cardiology, Hanoi Medical University, Vietnam (Q.N.N.).; 12 Department of Cardiology, Sarawak Heart Centre, Kota Samarahan, Malaysia (T.K.O.).; 13 Hero DMC Heart Institute, Dayanand Medical College and Hospital, Ludhiana, India (G.S.W.).; 14 Department of Cardiovascular Medicine, State Key Laboratory of Medical Genomics, Shanghai Key Laboratory of Hypertension, The Shanghai Institute of Hypertension, National Research Center for Translational Medicine at Shanghai, Ruijin Hospital, Shanghai Jiao Tong University School of Medicine, China (J.-G.W.).; 15 Division of Cardiology, Department of Medicine, Hong Kong Sanatorium & Hospital (Y.-T.A.W.).; 16 Division of Cardiology, Faculty of Medicine, Siriraj Hospital, Department of Medicine, Mahidol University, Bangkok, Thailand (N.W.).; 17 Cardiovascular Center and Division of Cardiology, Department of Internal Medicine, National Taiwan University Hospital and National Taiwan University College of Medicine, Taipei City (T.-D.W.).

**Keywords:** blood pressure, blood pressure monitoring, ambulatory, cardiovascular diseases, heart disease risk factors, hypertension

## Abstract

The second Asia Renal Denervation Consortium consensus conference shared information and developed updated recommendations for renal denervation (RDN). Current evidence confirms that RDN significantly reduces blood pressure across all metrics (office, home, and ambulatory) throughout 24 hours. Modern RDN approaches target the distal main renal artery and branches where nerves more closely approximate the vessel wall. Understanding renal artery anatomy is crucial; the main renal artery typically divides into anterior and posterior divisions as first-order branches, which further subdivide into second-order segmental arterial branches. Renal artery electrical stimulation shows promise as a procedural end point, with blood pressure response attenuation after successful RDN suggesting adequate denervation, though the optimal procedural end point remains to be established. The indication for RDN is resistant or uncontrolled hypertension, with high office, home, or 24-hour ambulatory blood pressure readings despite appropriate lifestyle modification and antihypertensive drug therapy. Preprocedure assessment includes comprehensive screening for secondary causes and detailed renal artery imaging. Checklists for preprocedure and postprocedure assessment are provided. Nocturnal hypertension and morning hypertension, which are common in Asia, are more closely associated with cardiovascular risk than daytime hypertension and are more difficult to control with current guideline-driven medication. Based on these Asian characteristics and RDN’s long-term durability, RDN should be considered an effective option for facilitating optimal 24-hour blood pressure control. Future research through real-world data collection will help determine ethnic differences in RDN response between Asians and Westerners and identify optimal candidates. In addition, studies are needed to evaluate RDN’s ability to prevent organ damage and cardiovascular events.

Transcatheter renal denervation (RDN) is an innovative treatment for hypertension that acts mechanistically by disrupting neural communication between the kidney and the brain. Early proof of concept trials^1–4^ were followed by multiple positive clinical trials that have evaluated the blood pressure (BP)–lowering effects of RDN.^5–15^ The considerable available body of evidence resulted in the US Food and Drug Administration approving both the PARADISE^TM^ ultrasound RDN and SYMPLICITY SPYRAL^TM^ radiofrequency RDN systems in 2023 for use in patients with inadequate BP control despite lifestyle modification and antihypertensive drug therapy. RDN had been previously approved for the treatment of uncontrolled hypertension in Europe, as well as many other countries worldwide. The current European Society of Hypertension^16^ guidelines and the European Society of Cardiology guidelines on hypertension both assigned a class II recommendation for RDN in patients with uncontrolled hypertension.^16,17^ Hence, RDN is now ready for clinical practice.

The first Asia Renal Denervation Consortium (ARDeC) consensus conference included Asian physicians actively performing RDN, who shared up-to-date information and regional perspectives, resulting in the consortium’s first RDN consensus document in 2020.^18^ As Asia now prepares for the broader introduction of RDN into clinical practice and the advances in science of RDN, the second ARDeC conference was convened in 2024 to discuss 7 important topics relating to the application of RDN in the context of Asian characteristics of hypertension. The topics include (1) BP-lowering effects of RDN; (2) evidence regarding the effects of RDN on organ damage and cardiovascular disease; (3) determinants of the effectiveness of RDN; (4) considerations relating to renal artery anatomy and sympathetic nerves; (5) denervation techniques; (6) indications and preparations for RDN; and (7) patient preference, shared decision-making, and clinical pathways. The key points from these discussions are summarized in this second ARDeC consensus document.

## BP-Lowering Effects of RDN

### Key Points

1. Transcatheter RDN safely and effectively reduces all BP metrics (office, home, and 24-hour ambulatory BP), irrespective of antihypertensive medication use.
2. In some sham-controlled trials of RDN, unexpectedly large reductions in BP were seen in the sham control group, which were possibly related to observed improved drug adherence, especially in the sham group.
3. RDN significantly reduces nighttime and morning BPs, which are more challenging to control with antihypertensive drug therapies and often remain poorly managed when treatment is guided by standard office BP measurements.
4. The BP-lowering effects of RDN persist for at least 10 years after the procedure.
5. The best BP metric to evaluate the long-term effects of RDN is morning home BP, followed by nighttime ambulatory or home BP, and then trough morning office BP (before antihypertensive drug dosing).
6. Given the Asian BP profile (where nocturnal hypertension and morning hypertension are common) and the fact that nighttime or morning BP provides a better indication of cardiovascular risk than daytime and office BP, RDN should be considered a reasonable option to help achieve optimal 24-hour BP control.

### Evidence From Sham-Controlled Trials

Multiple large, prospective, randomized, sham-controlled trials have reported on the safety and efficacy of RDN in patients with uncontrolled hypertension in both the presence and absence of concomitant antihypertensive drug therapy using various technologies (Table 1).^4,7–12,14,15,19^ Most trials used ambulatory BP monitoring metrics (24-hour BP or daytime ambulatory BP) as the primary end point. All 3 off-med trials using US Food and Drug Administration–approved radiofrequency or ultrasound RDN devices showed that RDN significantly reduced ambulatory BP compared with the sham control^8–10^ and the 3 on-med trials using US Food and Drug Administration–approved radiofrequency or ultrasound RDN devices, and RDN significantly reduced daytime or 24-hour ambulatory BP from baseline by 6.5 to 8.0 mm Hg.^7,11,13^ However, in 2 of these trials (REQUIRE [Renal denervation on Quality of 24-hr BP control by Ultrasound In Resistant hypertension]^13^ and SPYRAL HTN-ON MED [Study of Renal Denervation With the Symplicity Spyral™ Multi-electrode Renal Denervation System]^11^), there was also a significant reduction in ambulatory BP from baseline in the sham control group.

**Table 1.**
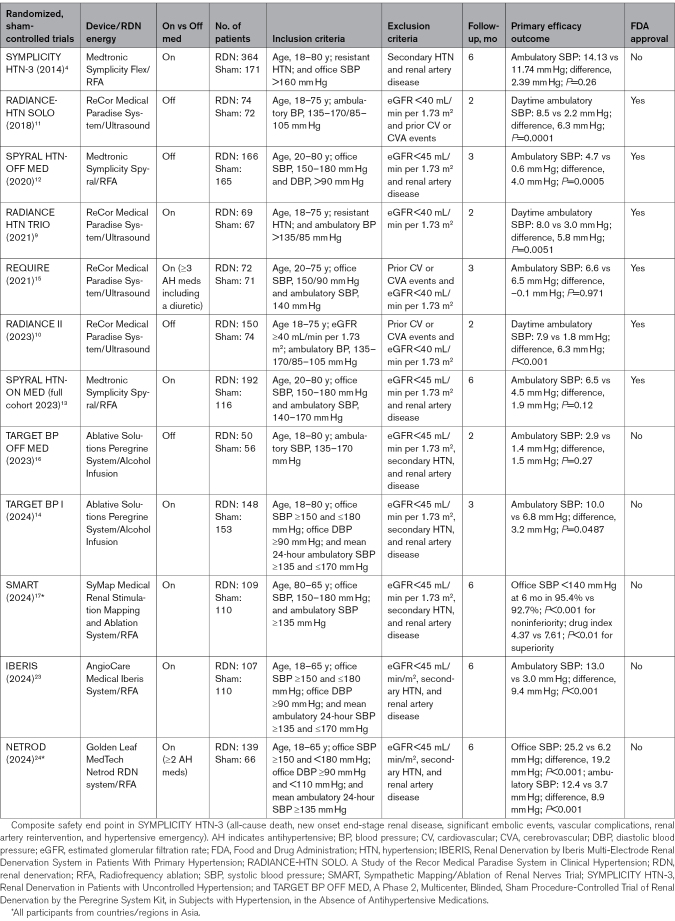
Summary of Clinical Trials of Percutaneous Transcatheter RDN

Clinical trial experience has consistently shown that changes in patient behavior during the primary follow-up period may have a significant impact on trial results.^20^ For example, analysis of data from the REQUIRE trial found that adherence to antihypertensive drug therapy was poor at baseline in the sham control group and improved after the procedure.^13^ A similar pharmacological dilution of the RDN treatment effect was observed in the SPYRAL HTN-ON MED expansion.^11^ The number of antihypertensive agents being used at 3 and 6 months after RDN was greater in the sham versus RDN group in both trials.

Recently, the results of 5 clinical trials, including 2 using ethanol injection RDN (TARGET BP OFF MED [A Phase 2, Multicenter, Blinded, Sham Procedure-Controlled Trial of Renal Denervation by the Peregrine System Kit, in Subjects with Hypertension, in the Absence of Antihypertensive Medications]^14^ and TARGET BP 1 [randomized, blinded, multi-center, international, sham-procedure controlled trial, comparing renal denervation with the Peregrine System Kit to the sham control group]^12^), 2 using radiofrequency ablation,^19,21^ and 1 using a mapping system for selected radiofrequency ablation (SMART [Sympathetic Mapping/Ablation of Renal Nerves Trial]^15^), have been published. The TARGET BP OFF MED trial did not find any significant difference in ambulatory systolic BP (SBP) at 2-month follow-up between the RDN and sham control groups, but medication burden at 12 months was lower in the RDN group.^14^ The TARGET BP I trial included medicated patients with uncontrolled hypertension and found a significant difference in 24-hour SBP at 3-month follow-up in the RDN versus control group (–3.2 mm Hg; *P*=0.0487).^12^

Three recent radiofrequency RDN trials (SMART,^15^ IBERIS [Renal Denervation by Iberis Multi-Electrode Renal Denervation System in Patients With Primary Hypertension],^21^ and NETROD^TM^^19^) were conducted exclusively in Asian (Chinese) populations. The SMART trial of renal nerve mapping/selective RDN in patients with uncontrolled hypertension in China^15^ applied a composite primary end point that included the proportion of patients achieving office SBP <140 mm Hg and the change in the composite index of antihypertensive drugs (both at 6-month follow-up). The results showed that patients randomized to the mapping/selective RDN group achieved a similar office SBP control rate compared with those in the sham control group despite using fewer antihypertensive medications. The other 2 radiofrequency RDN trials (IBERIS^21^ and NETROD^19^) treated patients with uncontrolled hypertension in China and reported a substantial and significant difference in 24-hour ambulatory SBP (approximately –9 mm Hg) in the RDN versus the control group at 6 months after the procedure (Table 1).

### Meta-Analyses and Systematic Reviews

The abundance and variety of rigorously designed and executed randomized controlled trials examining the safety and efficacy of RDN in different populations using different technologies have led to the recent publication of multiple meta-analyses.^22–25^ These analyses have consistently concluded that RDN significantly reduces office, home, and ambulatory BP measurements in patients with hypertension, both in the presence and absence of antihypertensive medication and between devices (Table S1).

### Beyond Sham-Controlled Trials: Other Studies of RDN in Asia

Besides Western populations, the safety and efficacy of RDN have also been studied in Asian populations. SYMPLICITY HTN Japan was the first randomized study to evaluate radiofrequency RDN in an Asian population.^26^ Despite being underpowered, the change from baseline in office SBP in the RDN group at 6 months (–16.6 mm Hg; *P*<0.001) was similar to that observed in the SIMPLICITY HTN-3 study although the change in SBP in the control arm (–7.9 mm Hg; *P*=0.117) was much smaller than that observed in the sham control group in SIMPLICITY HTN-3.

Registry data also confirm the impact of RDN in individuals from Asia. The Global SYMPLICITY Registry (GSR) is a prospective, all-comer, worldwide registry that evaluates the safety and effectiveness of radiofrequency RDN.^27^ A subanalysis of the GSR investigated RDN for the treatment of uncontrolled hypertension in South Korean patients.^28^ The mean change from baseline in office SBP was –19.4±17.2 mm Hg (*P*<0.001) at 6 months after RDN and –27.2±18.1 mm Hg (*P*<0.001) at 12 months. This greater reduction in BP at 12 months versus 6 months was not seen in the subset of White participants from the GSR. Progressively greater reductions from baseline in office BP in GSR Korea were also observed at 24 and 36 months post-procedure (–30.1±21.6 mm Hg [*P*<0.001] and –32.5±18.8 mm Hg [*P*<0.001], respectively). One potential explanation for the greater reductions in BP after RDN in Asian individuals in GSR Korea compared with Whites could be the intrinsically higher sensitivity to sympathetic modulation within the Asian population.^20^

GSR data have also been analyzed for the Taiwanese subpopulation^34^ including patients with refractory hypertension undergoing radiofrequency RDN. Both groups showed significant reductions in office SBP from baseline at 3 months (–15 to –21 mm Hg). Again, progressively greater reductions in office SBP versus baseline were observed over longer-term follow-up to 3 years after RDN.^29^

### Focus on the Reduction of Nighttime and Morning BPs

Control of BP during nighttime and the early morning period is critically important due to increased cardiovascular risk, especially risk of heart attack and stroke. Reduction in nighttime BP appears to be a key clinical effect of RDN. Multiple published reports consistently found a significant difference between RDN and sham control with respect to reductions in nighttime and morning BPs.^7–11,30^

Recently published hypertension guidelines recommended the measurement of nighttime BP because it is a stronger predictor of cardiovascular events compared with daytime BP. Also, individuals with a riser pattern of nighttime BP (ie, higher nighttime BP than daytime BP) or nondipper (less nocturnal BP dipping) are at higher cardiovascular risk than those with adequate nocturnal BP dipping.^31–34^ The nondipper and riser patterns of nighttime BP are associated with increased muscle sympathetic nerve activity,^35^ which has been shown to be reduced by radiofrequency RDN^36^ and indicating that RDN could be an effective approach to reducing nighttime BP in these individuals.^37–40^ Antihypertensive drug therapy may not be as effective in reducing nighttime BP because drug dosing is typically based on office BP readings in clinical practice.

Morning home BP is a stronger predictor of cardiovascular events compared with evening home BP in patients with hypertension, independent of office BP.^41,42^ Morning hypertension and an exaggerated morning BP surge are associated with elevated cardiovascular risk.^43^ Sympathetic nerve activity is increased in the morning compared with other times of the day, and RDN has been shown to significantly reduce morning ambulatory BP.^38–40^ A post hoc analysis of the SPYRAL HTN-ON MED trial also found that RDN attenuated morning BP surge.^44^

Morning home BP measured just before drug dosing could be the optimal metric to assess the BP-lowering effects of RDN (Figure S1). In the era of home BP–centered management of hypertension,^44–46^ morning home BP is ideally suited to evaluate the long-term effects of RDN on BP, and clinical evidence for the utility of validated nocturnal home BP measurements is growing.^47^

### Durability of BP Response

Data from long-term follow-up of multiple clinical trials and registries indicate that the BP-lowering effect of RDN is durable, as summarized in Table S2. Three-year follow-up data from 2 of the randomized sham-controlled trials of RDN showed persistent and significantly greater reductions from baseline in office and 24-hour ambulatory BP in the RDN group compared with sham control.^40,48^ Although there was no significant difference in the primary end point outcome between the RDN and standard treatment groups in the SYMPLICITY HTN Japan study,^26^ extended 3-year follow-up data indicated changes from baseline in office SBP/diastolic BP of – 32.8±20.1/–15.8±12.6 mm Hg (*P*<0.001).^49^

Several recent reports have followed local patient populations from early radiofrequency RDN clinical trials up to 8 to 10 years of follow-up. These trials have consistently demonstrated that the antihypertensive effects of RDN are enduring and may even increase over time despite no change or even slight reductions in the number of prescribed antihypertensive drugs.^50–54^ One single-center, single-arm, real-world study with a 10-year follow-up of RDN in China suggested that the procedure might be particularly beneficial for patients with elevated renin levels or high baseline SBP.^50^ Likewise, a recent meta-analysis found that there were clinically relevant BP reductions for up to 36 months after RDN based on randomized, sham-controlled trial data and for up to 10 years based on data from observational cohort studies.^55^ Preclinical studies have also suggested that the RDN treatment effect is durable. Indeed, the lack of myelination of the renal nerves, coupled with local fibrosis at the site of the lesion, limits anatomic and functional nerve recovery and results in durable axonal loss distal to the ablation site.^56^ Taken together, the preclinical and clinical results suggest that the need for repeat procedures following RDN may be minimal and that RDN may be considered a 1-time procedure.

### Adverse Events

Unlike drug therapy, RDN is a minimally invasive procedure and, thus, implies some risk to the patient. However, no differences were observed in the rate of adverse events after RDN compared with a sham procedure in the randomized controlled trials, and RDN is generally regarded as a safe procedure.^25^ The rate of renal artery stenosis requiring intervention following RDN is low,^57^ and long-term renal function does not seem to be adversely affected by RDN, even when the procedure is performed in patients with decreased kidney function.^58,59^

### Clinical Impact of RDN in Asia

#### Characteristics of Hypertension

In Asia, hemorrhagic stroke and nonischemic heart failure are common hypertension-related cardiovascular diseases, and the association between increases in BP and cardiovascular disease risk is stronger in Asians than in Westerners.^60,61^ However, the status of BP control in Asia varies markedly between countries/regions, with better reported control rates in Taiwan and South Korea, but suboptimal control in Japan.^62^

Among the distinguishing characteristics of Asian individuals are a large morning BP surge,^63^ early morning hypertension and nocturnal hypertension,^64^ and masked hypertension,^65^ which may be due to increased salt sensitivity and a high salt intake within the population.^60,61^ Higher salt intake in Asia increases the circulating blood volume and sympathetic tone and is likely to increase with mild obesity in young adults.^60^ Increased circulating blood volume may contribute to nocturnal hypertension, and increased sympathetic tone may also increase early morning hypertension and BP variability, resulting in uncontrolled hypertension. This is especially true for individuals from India, who have a resting heart rate that is about 5 bpm higher than that of individuals from other parts of Asia.^66^ These Asian-specific characteristics of hypertension suggest that RDN could be a reasonable option for improving hypertension management in the region.

#### Nocturnal Hypertension and Morning Hypertension

Nocturnal hypertension and morning hypertension are associated with the occurrence of cardiovascular events in Asia, especially for stroke.^32,33,41–43^ Thus, controlling nocturnal hypertension and morning hypertension is especially important in Asia.^67,68^ However, nearly half (45%) of Japanese patients with hypertension who are being treated with ≥3 antihypertensive medications have uncontrolled nocturnal hypertension, and just over half (55%) have uncontrolled early morning hypertension (Figure S2).^69^

**Figure 1.**
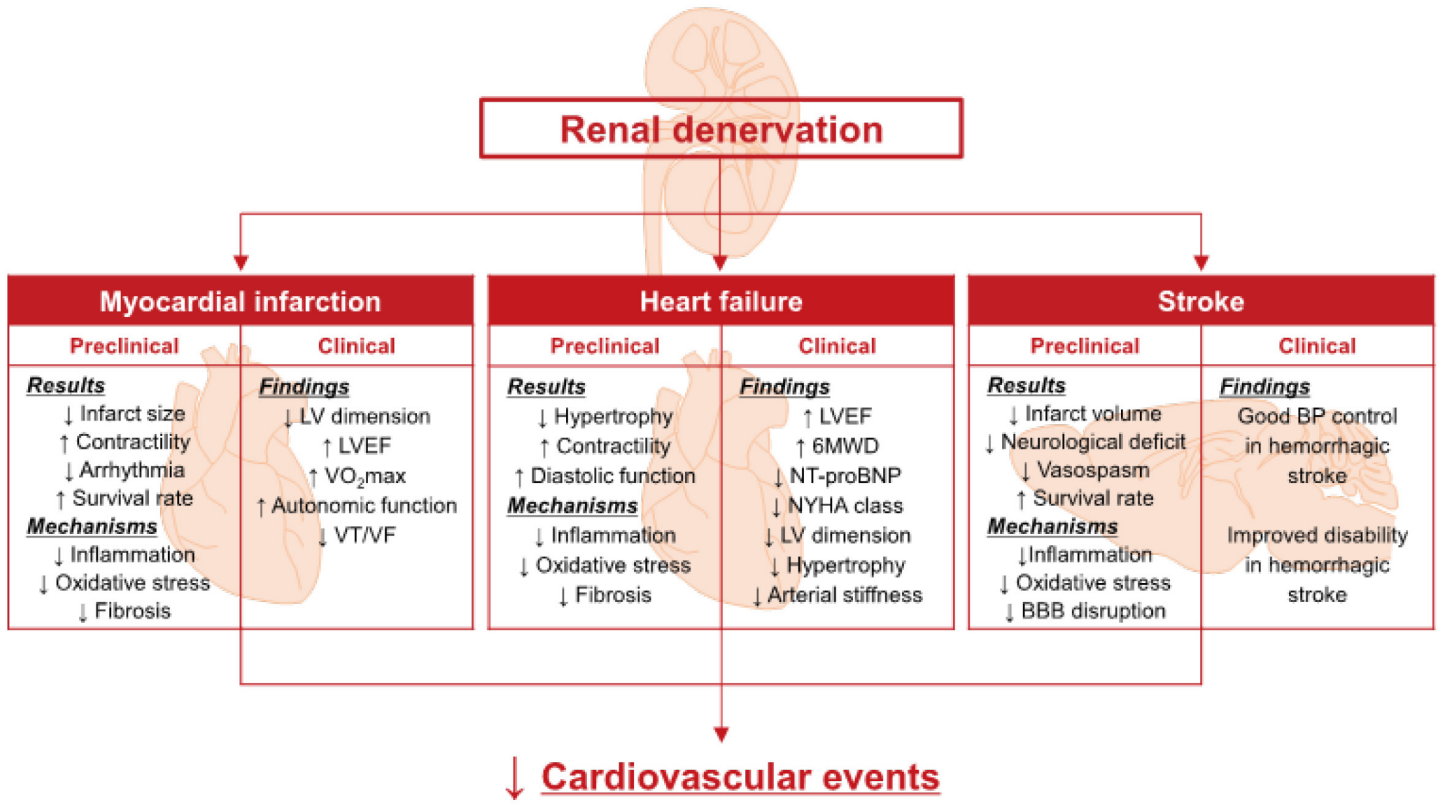
**Effects of renal denervation on organ damage and cardiovascular diseases.** BBB indicates blood-brain barrier; BP, blood pressure; LV, left ventricular; LVEF, left ventricular ejection fraction; 6MWD, 6-minute walk distance; NYHA, New York Heart Association; VF, ventricular fibrillation; VO2max, maximal oxygen uptake; and VT, ventricular tachycardia. Used with permission from Katsurada K and Kario K. Hypertens Res 2024.

Uncontrolled nocturnal hypertension and early morning hypertension represent one of the greatest challenges for future hypertension practice,^70^ and RDN should be considered in people with uncontrolled morning hypertension and residual nocturnal hypertension treated with antihypertensive pharmacotherapy in clinical practice.^68^ RDN may be effective in reducing target-organ damage and the subsequent risk of cardiovascular disease (Figure 1).

## Factors Influencing RDN Effectiveness

### Key Points

1. Determinants of RDN effectiveness include patient characteristics, biomarkers, procedural variables, and parameters of invasive/provocative testing.
2. Absence of these predicting parameters does not exclude patients undergoing RDN, especially for high-risk patients with refractory hypertension or resistant hypertension with evidence of target organ damage.
3. Asia has a higher prevalence of salt-sensitive patients, nocturnal hypertension patients, and morning hypertension patients, which are all related to responsiveness to RDN.
4. RDN should be offered early in the treatment pathway in patients with features suggestive of RDN effectiveness.

Patients with high basal sympathetic activity or neurogenic hypertension may have more pronounced BP reduction after RDN than patients with hypertension from other causes. Direct or indirect measurement of sympathetic nerve activity, such as muscle sympathetic activity, norepinephrine spill over, plasma renin activity, impaired baroreflex sensitivity, or high basal heart rate, can predict a greater reduction of BP after RDN.^71–75^ However, sympathetic nerve activity measurement is not practical in daily clinical practice. Likewise, discontinuing prescribed antihypertensive medications, which may be necessary to observe basal sympathetic activity, could be dangerous in patients with refractory hypertension.

Invasive and noninvasive measurements of arterial stiffness have also shown an association with RDN effectiveness.^76–81^ Patients with lower pulse wave velocity, associated with higher arterial elasticity and distensibility, have more pronounced BP reduction after RDN. Common hypertension phenotypes associated with renal sympathetic activation include mild essential hypertension, isolated systolic hypertension in the young, and obesity hypertension.^82^ In addition, salt sensitivity is a common pathophysiology of hypertension in Asia.^83^ High salt sensitivity index, characterized by heart rate ≥70/min and mean arterial pressure dipping ≤10%, was shown to be associated with greater BP reduction in RDN.^84^

**Figure 2.**
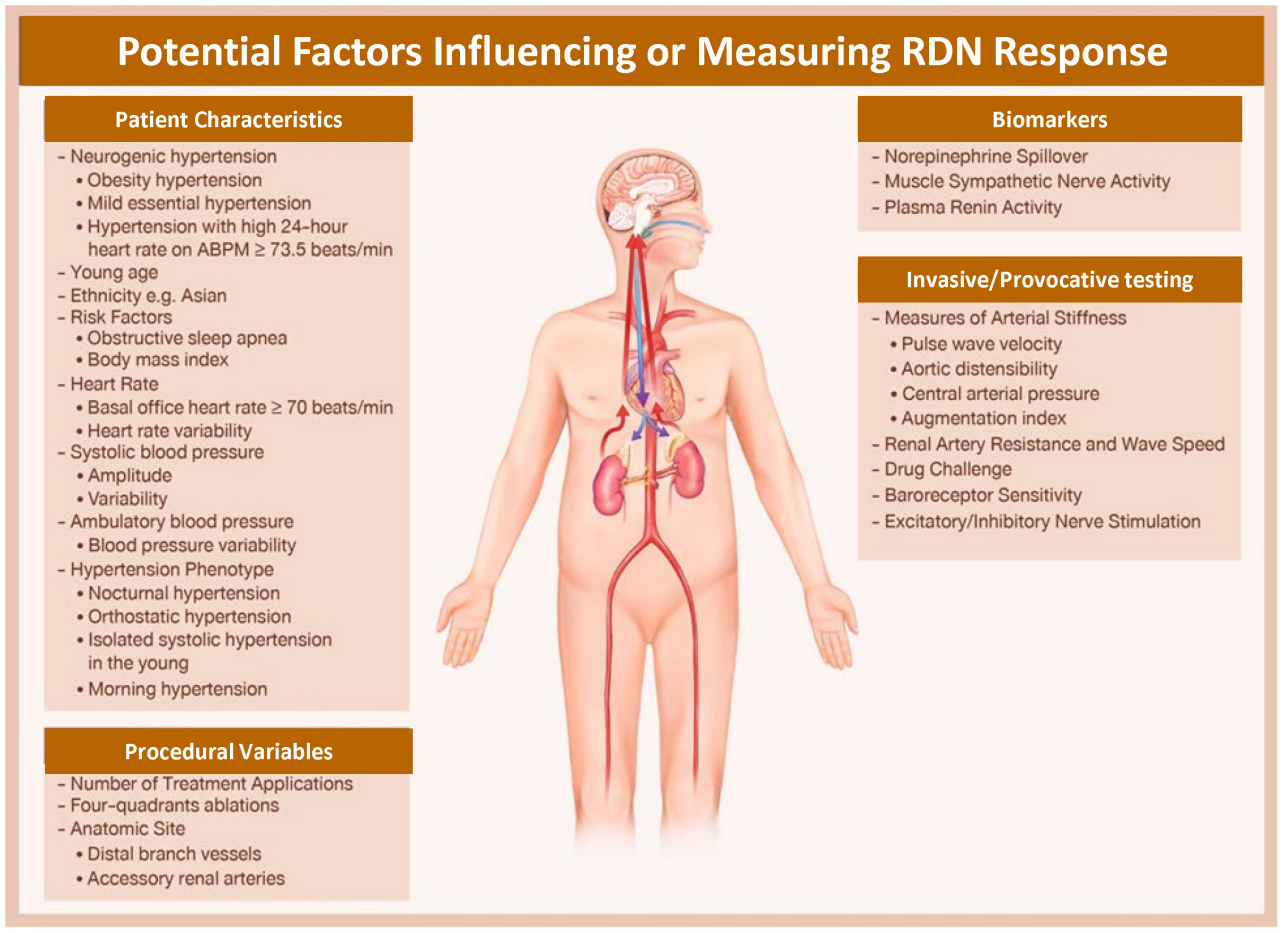
**Potential factors influencing or measuring renal denervation (RDN) response.** ABPM indicates ambulatory blood pressure monitoring.

No practical method is currently available to verify complete nerve destruction during the procedure. Therefore, optimization of RDN procedural techniques to ensure complete ablation is critically important. Performance of full circumferential ablation and completely treating the renal artery, including the main, branch, and accessory arteries, when present, is important to maximize RDN effectiveness.^85–87^ Intraprocedural blunting of BP elevation response elicited by radiofrequency renal artery stimulation may indicate complete denervation and has shown good association with BP response after RDN.^88,89^ Prospective evaluation is still required to identify a clear and reliable metric of complete denervation. Potential factors influencing or measuring RDN responses are summarized in Figure 2.

## Anatomy of the Renal Artery and Sympathetic Nerve

### Key Points

1. The kidney is composed of 5 segments, each supplied by a segmental artery.
2. The main renal artery typically divides into anterior and posterior divisions as first-order branches, which then further subdivide into segmental arteries as second-order branches.
3. The posterior division usually points downward perpendicular to the main renal artery and then turns 90° toward the posterior segment of the kidney.
4. Modern RDN approaches have evolved to target the distal main renal artery and branches where nerves more closely approximate the vessel wall.
5. Significant sympathetic nerves are present around accessory renal arteries. Therefore, accessory arteries should be treated if they are of sufficient size.
6. The ratio of sympathetic efferent to afferent nerves increases from proximal to distal main renal artery, with efferent nerves comprising over 90% in distal segments.
7. It is still controversial whether parasympathetic nerves exist in the kidney. Given the absence of these cholinergic nerves in the renal parenchyma, there is no evidence showing that the renal cholinergic nerves play any role in systemic BP modulation.

### Renal Artery Anatomy and Nerve Distribution

**Figure 3.**
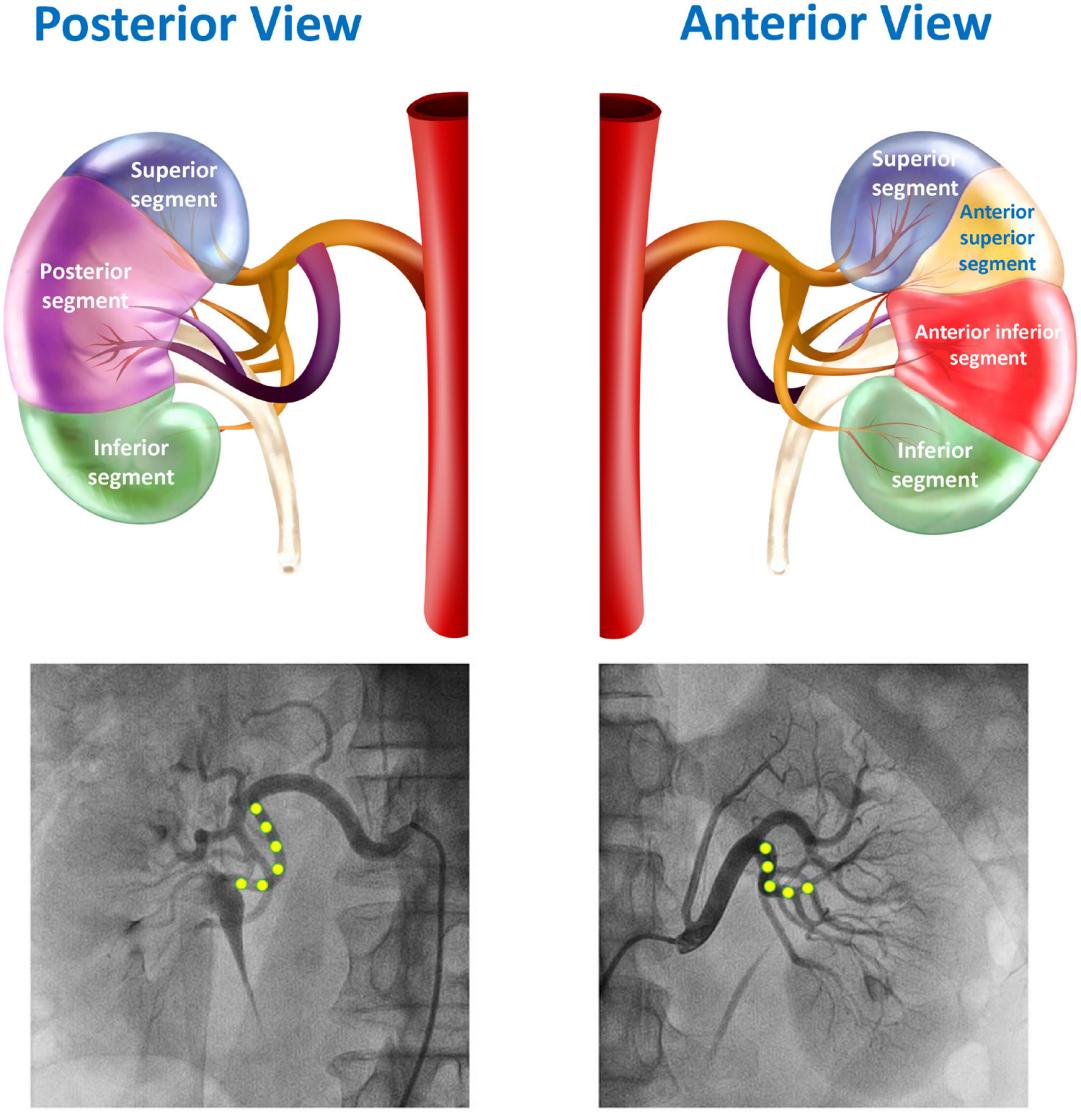
**Anatomy of the kidney and renal arterial system.** The kidney is anatomically divided into 5 perfusion segments, each supplied by a specific segmental artery. The main renal artery (brown) typically divides into anterior (brown) and posterior (purple) divisions as first-order branches, which are separated by the ureter. The anterior division subdivides into 4 segmental arteries (the superior, anterior superior, anterior inferior, and inferior segmental arteries). The posterior division is usually oriented downward and perpendicular to the main renal artery. The posterior division supplies one segmental artery (the posterior segmental artery). Renal artery anatomy by angiography is shown below the kidney schematic: anterior/posterior (yellow circles) divisions (first-order branches) and segmental arteries (second-order branches).

The kidney itself is anatomically divided into 5 perfusion segments (Figure 3), each supplied by a specific segmental artery.^90^ The main renal artery typically divides into anterior and posterior divisions as first-order branches, which then further subdivide into segmental arteries as second-order branches.^90^ The anterior division receives around 75% of the total renal blood flow and typically subdivides into 4 segmental arteries (the superior, anterior superior, anterior inferior, and inferior segmental arteries). In contrast, the posterior division receives the remaining 25% of blood flow and supplies one segmental artery (the posterior segmental artery).^91^ The posterior division is usually oriented downward and perpendicular to the main renal artery and then angles 90° toward the posterior segment of the kidney.

A clear anatomic understanding of the renal arterial perfusion system is crucial because renal nerves course farthest from the main renal artery and become closest to the vessel wall distal to the primary bifurcation. It was estimated that 75% of nerve fibers were within 5 mm of the distal main renal artery lumen, suggesting that achieving an ablation depth of 5 mm could affect over 80% of nerve fibers. Likewise, the majority of nerves are within 3 mm of the arterial lumen about the postbifurcation branches.^92^ Both animal and human studies have shown that performing RDN in the main artery plus its branches results in greater reductions in renal norepinephrine levels and BP compared with RDN in the main artery alone.^93,94^

Furthermore, late-arriving nerves often bypass the main renal artery and converge on the branches. In one study,^95^ late-arriving nerves were found in 73% of right kidneys and 53% of left kidneys. Moreover, 27% of these late-arriving nerves joined at or beyond the segmental branches (second-order branches) and were, thus, termed very late-arriving nerves. In addition, accessory renal arteries, present in ≈30% of individuals, are anatomically and functionally similar to those surrounding the main renal arteries.^95^ Treatment of distal main and accessory arteries (where vessel caliber permits) may be necessary for complete denervation, as studies have linked accessory artery treatment to the magnitude of BP reduction.^96^

## Indications and Assessments for RDN

### Key Points

1. The indications for RDN require implementation of lifestyle modifications and antihypertensive medications, along with uncontrolled out-of-office BP demonstrated by either ambulatory or home BP measurements.
2. The ARDeC recommends that ambulatory BP monitoring, after directly observed intake of antihypertensive medications if pharmacologically treated, should be conducted in all patients planned to be treated by RDN because it would provide the most comprehensive evidence regarding the BP-lowering efficacy.
3. The shared decision-making process can be initiated at the earlier stage of preprocedure assessment to allow more time to consider or consult.
4. RDN should not be performed in patients with untreated endocrine hypertension.
5. Thin-slice abdominal computed tomography (CT) with contrast is essential for identifying the anterior and posterior divisions of the main renal artery, which are separated by the ureter.
6. RDN should be performed at sites at least 5 mm away from any abnormal renal artery anatomy, including a preexisting stent.

### Indications

As the third pillar of the antihypertensive treatment armamentarium, the updated indications, additional compelling indications, and contraindications of RDN recommended by the ARDeC task force are shown in Table 2.

**Table 2.**
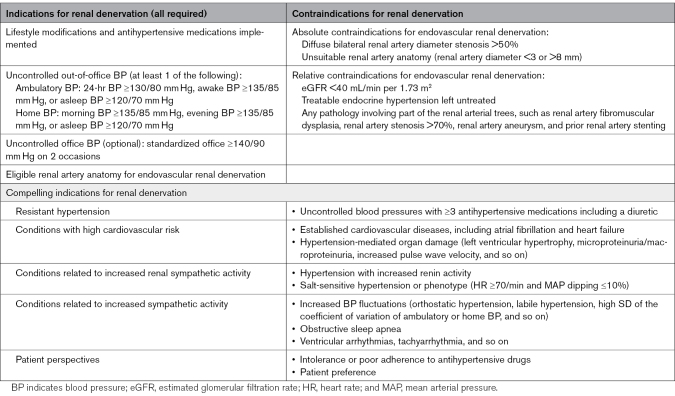
Indications and Contraindications for Renal Denervation

The indications for RDN require implementation of lifestyle modifications and antihypertensive medications, along with uncontrolled out-of-office BP demonstrated by either ambulatory or home BP measurements. Ambulatory monitoring requires 24-hour BP ≥130/80 mm Hg, awake BP ≥135/85 mm Hg, or asleep BP ≥120/70 mm Hg. For home measurements (average of 4- to 7-day measurements with the first day measurement discarded),^97^ morning or evening BP ≥135/85 mm Hg or asleep BP ≥120/70 mm Hg qualifies (Table 2). This recommendation is based on the consistent BP-lowering efficacy of RDN across the entire spectrum (from drug-naive to resistant) of patients with hypertension^98^ and is in line with the approval statement of the US Food and Drug Administration.^99^ The advantages of out-of-office BP measurements, compared with routine office BP, in hypertension management have been emphasized in recent European and Taiwanese hypertension guidelines.^17,46^ The task force recommends that ambulatory BP monitoring, after directly observed intake of antihypertensive medications if pharmacologically treated, should be conducted in all patients planned to be treated by RDN because this strategy is supported by the most comprehensive evidence regarding the BP-lowering efficacy.

The task force also identified compelling indications where RDN may be particularly beneficial or where it addresses specific patient needs (Table 2). Four main categories, which are based on the severity of hypertension, inherent cardiovascular risk, pathophysiology of hypertension phenotype, and patient preference, are identified. Resistant hypertension, defined as uncontrolled office and out-of-office BP despite ≥3 antihypertensive medications including a diuretic, represents a key indication, given that the BP-lowering effect conferred by RDN is directly related to baseline systolic BP.^100^ High cardiovascular risk conditions, including established cardiovascular diseases, atrial fibrillation and heart failure with preserved or reduced ejection fraction,^101^ or hypertension-mediated organ damage such as left ventricular hypertrophy, are additional compelling indications.

Conditions related to increased renal sympathetic activity, including hypertension with increased renin activity^72^ and salt-sensitive hypertension (characterized by heart rate ≥70/min and mean arterial pressure dipping ≤10%),^84^ are recommended as compelling indications. Conditions related to increased systemic sympathetic activity such as orthostatic hypertension and obstructive sleep apnea also represent compelling indications.^18^

From a patient perspective, antihypertensive drug intolerance or poor adherence to antihypertensive medications and patient preference after appropriate shared decision-making and counseling are important considerations. Future research leveraging trial codesign and patient lived experience is required to better understand the role of patient preference and shared decision-making.

Absolute contraindications preclude appropriate energy conveyance by RDN in certain cases (Table 2). These include diffuse bilateral renal artery diameter stenosis >50%, which usually occurs in patients with advanced CKD, and unsuitable bilateral renal artery anatomy. Relative contraindications include estimated glomerular filtration rate <40 mL/min per 1.73 m^2^, untreated endocrine hypertension, and any pathology involving part of the renal arterial trees, including renal artery fibromuscular dysplasia, renal artery stenosis, and renal artery aneurysm. In the latter cases, RDN should be performed at least 3 months following stent implant and at locations at least 5 mm away from any abnormal anatomy, including the stent.

### Preprocedure Assessment

**Figure 4.**
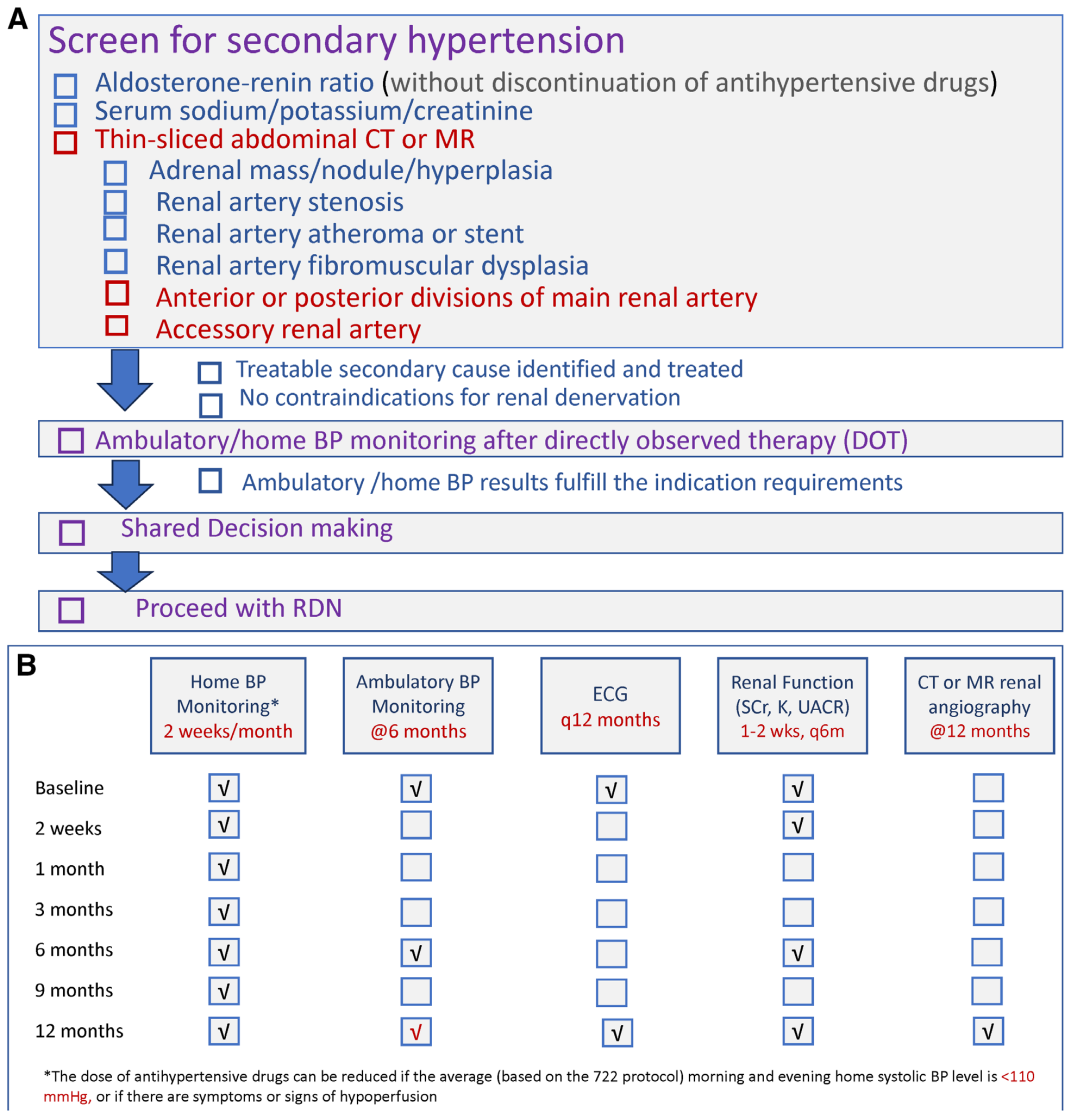
**Pre- and post-procedure assessment tools.** Clinical assessment before and after renal denervation (RDN) helps ensure optimal patient outcomes. **A**, Pre-RDN assessment checklist. **B**, Post-RDN assessment checklist. BP indicates blood pressure; CT, computed tomography; and MR, magnetic resonance.

A systematic multidisciplinary evaluation before RDN helps ensure optimal patient selection and outcomes. Understanding both the clinical assessment requirements and detailed renal anatomy is crucial for procedural success. The checklist for preprocedural assessment is provided (Figure 4A).

The preprocedure evaluation begins with a comprehensive screening for secondary causes of hypertension. This includes biochemical and imaging evaluation for various endocrine hypertension such as primary aldosteronism, Cushing syndrome, pheochromocytoma, thyroid dysfunction, and hyperparathyroidism.^102^ The task force recommends that the aldosterone-renin ratio measurement could be performed without discontinuing antihypertensive drugs,^103,104^ thus simplifying implementation. RDN should not be performed in patients with untreated endocrine hypertension (Table 2).

Accessory renal arteries, present in approximately one-third of individuals, also require consideration.^95^ Regular slice thickness in computed tomography scans may not recognize the presence of an accessory renal artery due to limited spatial resolution. In this regard, thin-slice abdominal computed tomography is recommended. The preprocedure evaluation should be followed by documentation of out-of-office BP status after directly observed intake of antihypertensive medications. Either ambulatory (preferred) or home BP monitoring is recommended.

After confirming patient eligibility, the final step is to initiate the shared decision-making process. The major goals are to engage the patient in discussions about the procedure, including its potential benefits, risks, and alternatives, and to educate the patient about the expected outcomes and the importance of continued lifestyle modifications and medication adherence. The shared decision-making process can be initiated during the earlier stage of preprocedure assessment to allow more time to consider or consult.

### Postprocedure Monitoring and Follow-Up

Following RDN, a structured follow-up protocol is essential to monitor outcomes, ensure safety, and optimize patient care. The task force provides a comprehensive post-RDN assessment checklist (Figure 4B).

Early postprocedure monitoring focuses on access site complications and renal function. While same-day discharge is feasible,^105^ patients should be monitored based on institutional protocols for femoral access procedures.

BP response assessment typically begins at 1 to 2 months post-procedure. This timing allows for the effect of denervation to manifest while providing early feedback on treatment success. Ambulatory BP monitoring is recommended at 6 and 12 months post-RDN when the full BP effect should be apparent. Initiation of regular home BP monitoring is recommended as early as possible, given its relative ease of implementation. The dose of antihypertensive drugs can be reduced if the 4-to-7-day averaged morning and evening home systolic BP level is <110 mm Hg or if there are symptoms or signs of hypoperfusion.^46^

Response to RDN varies among patients, with approximately two-thirds to four-fifths showing clinically meaningful BP reductions.^11,19,106^ Current evidence indicates that higher baseline systolic BP may predict greater response to RDN though individual results remain difficult to predict.^100^

The success of RDN as a durable BP-lowering treatment modality hinges on meticulous patient preparation and comprehensive postprocedure follow-up. By adhering to the standardized protocols outlined in the ARDeC Consensus, health care providers can ensure patient safety, optimize outcomes, and contribute to the evolving field of RDN.

## Patient Preference, Shared Decision-Making, and Clinical Pathway

### Patient Preference

Medication adherence is often low among younger patients with hypertension, particularly when they are prescribed multiple drugs^107^ Furthermore, patients’ perspectives and prioritization of the management of their illness may differ from that of the managing clinician. This is particularly true for RDN, where studies have shown that as many as a third of patients verbally opt for RDN as a possible alternative to initiating or increasing life-long drug therapy for managing their BP. Notably, even among those patients who were already treated with ≥1 antihypertensive medication, a third were also willing to opt for RDN.^108–110^ These findings indicate that patients’ preference for RDN is not just based on pill burden or the severity of their hypertension.^109,111^ Hence, the preference to improve BP control, without having to resort to increased medication burden, needs to be discussed in a shared decision manner with the patient, highlighting the anticipated benefits, safety, cost, and acceptance of the procedure.

### Clinical Pathway

Patients with hypertension who may potentially benefit from RDN need to be carefully identified. The clinical pathway will include identifying patients with uncontrolled hypertension, confirming that it is truly uncontrolled and not white coat hypertension, with the use of out-of-office BP measurements, checking adherence, and excluding secondary causes (Figure 4A).

Ideally, a formal referral pathway should be established, as well as a prescribed, clinical follow-up regimen for patients post-RDN, where BP changes are monitored and where changes in medication may be required, and for monitoring renal function and screening for possible complications of the procedure (Figure 4B). Last, it is also important to manage the referring doctor’s expectations for both the timing and magnitude of the BP-lowering response.

## Asian Perspectives of RDN

The most recent hypertension guidelines of several Asian countries and regions are generally positive or keen regarding the use of RDN for the treatment of hypertension. In the 2022 Taiwan hypertension guidelines, RDN was recommended for the treatment of hypertension in patients at high cardiovascular risk, such as those with resistant or masked uncontrolled hypertension, established atherosclerotic diseases, intolerant or nonadherent to antihypertensive drugs, or features indicative of neurogenic hypertension after careful clinical and imaging evaluation, with a class of recommendation IIa and level of evidence of B (Table S3).^46^ In the most recently published 2024 Chinese guidelines, RDN was recommended for the treatment of hypertension in patients with drug-resistant hypertension or poor adherence to medication after excluding secondary hypertension with a class of recommendation of IIb and level of evidence of B.^112^ In the meantime, the Chinese guidelines also emphasized that RDN should be performed in hospitals with extensive experience in the diagnosis and treatment of hypertension and the capacity to differentiate the causes of secondary hypertension. The Hypertension Cardiovascular Outcome Prevention and Evidence in Asia Network recommends a home BP–centered approach to hypertension management, focusing on morning and nocturnal hypertension,^68^ and RDN as one of the options that could be used to help achieve this goal.^68^ In the consensus statement on RDN by the Joint Committee of the Japanese Society of Hypertension, the Japanese Association of Cardiovascular Intervention and Therapeutics, and the Japanese Circulation Society, uncontrolled nocturnal hypertension and morning hypertension are specified as indications for RDN.^113^

All these guidelines reflect a pragmatic attitude in the use of this device-based therapy for the treatment of hypertension. One consistently identified key requirement is the careful and informed selection of patients who may have a positive BP response and, hence, are most appropriate for this treatment. A prospective clinical trial of RDN targeting morning hypertension in Asia is needed to help identify subsets of patients who are most likely to benefit from this therapy.

## Evidence Gaps and Future Goals of ARDEC

RDN has emerged as a promising interventional approach for treating hypertension, with demonstrated safety and efficacy in lowering BP across various patient populations. However, several important evidence gaps remain that need to be addressed. Long-term data on cardiovascular outcomes in Asian populations remain limited although the durability of BP-lowering effects for up to 10 years has been demonstrated.^54^ The identification of reliable predictors of treatment response remains challenging although periprocedural renal artery stimulation seems promising and is being investigated in Asia. In addition, the role of RDN in specific Asian hypertension phenotypes, such as salt-sensitive hypertension and morning/nocturnal hypertension, requires further investigation. Cost-effectiveness data specific to Asian health care systems are limited, which impacts reimbursement decisions and widespread adoption.^114^

Looking ahead, key goals for ARDeC include establishing and promoting standardized protocols for patient selection and procedural techniques across Asia, with particular focus on Asian-specific characteristics and phenotypes (Table S4). There is also a need to conduct dedicated trials examining the effectiveness of RDN in treating nocturnal hypertension and morning hypertension, which are particularly prevalent in Asian populations. The ARDeC consortium aims to develop clear guidance on integrating RDN into existing hypertension treatment pathways while considering regional health care system differences. Building a comprehensive Asian registry to collect real-world data on long-term safety, efficacy, and cardiovascular outcomes will be crucial. Fostering collaboration between centers to share expertise and standardize practices across the region also remains a priority. Success in addressing these goals will be essential for optimizing the role of RDN in hypertension management across Asia and ensuring this therapeutic option benefits those patients most likely to respond favorably to the treatment.

## ARTICLE INFORMATION

### Acknowledgments

Editing assistance was provided by Doug Hettrick, PhD, and Colleen Gilbert, PharmD, employees of Medtronic. Drs Gilbert and Hettrick provided editorial language and syntax support and did not contribute to the content of the consensus document. K. Kario and T.-D. Wang were responsible for the conception and design of the consensus, drafting the article, providing critical review and revision of the article, and the decision to submit the article. A.A. Alkatiri, J. Aoki, W.A. Wan Ahmad, Y.-C. Chia, J.N.M. Cruz, Y.-H. Lee, S.T. Lim, C. Lu, Q.N. Nguyen, T.K. Ong, G.S. Wander, J.-G. Wang, Y.-T. A. Wong, and N. Wongpraparut were responsible for drafting the article, providing critical review and revision of the article, and the decision to submit the article.

### Source of Funding

The Asian Renal Denervation Consortium meeting was supported by unrestricted educational funding from Medtronic. However, the meeting agenda, the decision to produce this consensus, the outline, and the drafting and revision of the consensus were all solely performed by the Asia Renal Denervation Consortium members with no input from the funder.

### Disclosures

K. Kario received lecture fees from Medtronic Japan Co. T.-D. Wang received honoraria from Medtronic Taiwan Co. Y.-H. Lee received honoraria from Medtronic and travel support from Boston Scientific, Medtronic, and Abbott. Y.-C. Chia received grant support from Viatris and Medtronic and received honoraria from Medtronic, Inc. S.T. Lim received travel/educational support for conference attendance from Medtronic, Asahi-Intecc, Orbus-Neich, AlviMedica, Terumo, Abbott Vascular, Boston Scientific, Biosensors, and Elixir; all honoraria received were donated to the National Heart Center Endowment Fund. The other authors report no conflicts.

### Supplemental Material

Tables S1–S5

Figures S1 and S2

Effects of RDN on Organ Damage and Cardiovascular Diseases

Renal Denervation Techniques

## Supplementary Material
