# Supplementary material for: Renal Denervation in Asia: 2025 Asia Renal Denervation Consortium (ARDeC) Consensus Statement Endorsed by the Hypertension Cardiovascular Outcome Prevention and Evidence in Asia (HOPE Asia) Network
Source: Hypertension. 2025 Dec 2;83(1):6–22. doi: 10.1161/HYPERTENSIONAHA.125.25333 (PMC12711283; doi:10.1161/HYPERTENSIONAHA.125.25333)
Supplement: Supplementary file 1 [file hyp-83-006-s001.pdf]

Supplemental Material

Renal Denervation in Asia: 2025 Asia Renal Denervation Consortium (ARDeC) Consensus Statement endorsed by the Hypertension Cardiovascular Outcome Prevention and Evidence in Asia (HOPE Asia) Network

Kazuomi Kario, Amir Aziz Alkatari, Jiro Aoki, Wan Azman Wan Ahmad, Yook-Chin Chia, Jose Nicolas M. Cruz, Ying-Hsiang Lee, Lim Soo Teik, Chengzhi Lu, Quang Ngoc Nguyen, Tiong Kiam Ong, Gurpreet S Wander, Ji-Guang Wang, Yiu-Tung Anthony Wong, Nattawut Wongpraparut, Tzung-Dau Wang

EFFECTS OF RDN ON ORGAN DAMAGE AND CARDIOVASCULAR DISEASES

Key points

1. RDN improved cardiac remodelling and reduced left ventricular hypertrophy beyond its BP reduction effects.
2. RDN improved the arrhythmia substrate and might become potential antiarrhythmic therapy.
3. RDN effectively reduces BP with no adverse effect on renal function.
4. There is no clear evidence of BP-independent beneficial metabolic effects of RDN.

Despite the lack of prospective outcome data, BP reduction following RDN may improve cardiovascular outcome. For example, increased time in the therapeutic BP range might protect or delay progression of target organ damage.[1] Notably, a comparison of actual cardiovascular event rates at 3 years from the GSR with a modelled projection based on published meta regression analysis of the hypertensive population showed significant absolute reductions in major adverse cardiovascular events over 3 years compared with the projected control (8.6±0.7% observed vs. 11.7±0.9% for projected control; $P<0.01$). These modelled reductions were primarily due to reduced stroke incidence.[2] RDN is also expected to reduce the onset and progression of cardiovascular diseases including myocardial infarction, heart failure, and stroke in clinical practice (**Figure 1**).[3] However, a clinical need exists to further validate the potential impact of RDN on cardiovascular outcomes.

Cardiac Protective Effects

In hypertensive patients, treatment with RDN resulted in improvements of cardiac indices reflecting adverse remodeling, especially left ventricular mass index and left ventricular ejection fraction, but not indices of diastolic function in meta-analyses.[4-7] In patients with heart failure with reduced ejection fraction, RDN can improve left ventricular structure and function irrespective of changes in BP.[8] BP was substantially reduced, left ventricular diastolic function improved and left ventricular mass regression was observed in several studies of patients with resistant.[9–11]

A meta-analysis on 681 patients with hypertension from 16 studies showed that RDN could reduce office heart-rate (-1.9 beats/min, $P<0.001$), 24-h heart rate, and daytime heart rate, but not nighttime heart rate, and the reduction of heart rate was highly related to SBP decrease.[12] The AFFORD study showed that stand-alone RDN did not significantly reduce atrial fibrillation burden up until 3 years of follow-up.[13] However, single center, randomized, sham-controlled RDPAF pilot trial showed that that RDN could reduce the occurrence of atrial arrhythmias in patients with hypertensive heart disease. Additionally, the combination of RDN with pulmonary vein isolation in patients with paroxysmal atrial fibrillation and hypertension significantly increased the likelihood of freedom from atrial fibrillation at 12 months compared with catheter ablation alone. Likewise, RDN combined with pulmonary vein isolation, compared with pulmonary vein isolation alone, reduced AF burden and antiarrhythmic drug usage in the SYMPLICITY-AF study[14] and a meta-analysis of 711 hypertensive patients with atrial fibrillation from 7 trials favored the combination of RDN and pulmonary vein isolation.[15] Similarly, a meta-analysis on 121 patients with ventricular tachycardia from 7 studies showed that RDN can significantly reduce implantable cardiac defibrillator therapies, ventricular arrhythmias episodes, anti-tachycardia pacing episodes and shocks.[16] These data indicated that

RDN can improve the arrhythmia substrate and might become a potential adjunctive antiarrhythmic therapy.

Renal Protective Effects

Several meta-analyses support the potentially positive effects of RDN on renal function. A meta-analysis on 2381 hypertensive patients from 48 studies showed no significant change in estimated glomerular filtration rate (eGFR) after a mean follow-up of 9 months.[17] Another meta-analysis on 226 patients with chronic kidney disease (CKD) from 11 prospective trials showed that RDN can interrupt natural CKD progression based on serial eGFR and serum creatinine changes up to 2 years of follow-up.[18] Furthermore, a meta-analysis on 238 patients with CKD and resistant hypertension revealed that RDN significantly decreased urinary albumin excretion at 3 and 6 months compared with baseline.[19] Whereas a sub-analysis of the SYMPLICITY HTN-3 found RDN did not reduce albuminuria or proteinuria in patients with CKD with insufficient antihypertensive effects.[20] Some studies have also shown reduced renal resistive index [21] [89] as well as increased vessel diameter and blood flow velocity.[22] RDN likely improves proteinuria and albuminuria through its antihypertensive effects.[23] A report of long-term outcomes in patients who are on dialysis found no adverse effects on dialysis tolerance as long as 4 to 7 years post-RDN.[24] These data suggest that RDN does not worsen renal function and further investigation is required to determine the role of RDN in improving or preserving renal function.[23]

Beneficial Metabolic Effects

A meta-analysis on 2245 patients from 19 studies showed that no significant changes after RDN in fasting glucose, insulin level, hemoglobin A1C, insulin resistance, total cholesterol and low-density lipoprotein cholesterol during follow-up ranged from 3 to 24 months.[25] While there were

inconsistent results regarding the effects of RDN on adipokines[26–28], body weight is likely unchanged after RDN throughout studies in patients with resistant hypertension.

RENAL DENERVATION TECHNIQUES

Key points

1. Radiofrequency, ultrasound, and alcohol-based devices have been tested in several randomized trials.
2. These devices should be applied appropriately according to patient characteristics and the anatomy of the renal artery.
3. Both renal artery electrical stimulation and trans-vascular pacing of aorticorenal ganglia can elicit blood pressure changes and renal artery vasoconstriction, which might serve as procedural endpoints for RDN.
4. The BP response to renal artery electrical stimulation is dynamic and can change not only during stimulation but also after stimulation has ceased. It is recommended to continue BP monitoring for at least 3 minutes following the discontinuation of electrical stimulation to obtain the whole profile.

Renal Denervation Techniques

Multiple distinct technologies have been evaluated for safety and efficacy for percutaneous RDN. Radiofrequency, ultrasound, and alcohol injection-based devices have been tested in randomized trials. Each technology has specific features **(see Supplement Table 2)**.[29-31]

The multielectrode Symplicity Spyral™ catheter (Medtronic, Inc) is a representative radiofrequency-based RDN system. The four electrodes on the catheter are positioned in a helical pattern to apply radiofrequency energy circumferentially in all four quadrants of the renal artery and branch vessels.[32] The total number of ablations performed within a segment is determined by the local vessel anatomy, starting distally and proceeding proximally without overlapping treatment zones. Ablation of accessory renal arteries, when present, is recommended when the diameter ranges from 3 mm to 8 mm. Each ablation time is 60 seconds. Mean lesion penetration depth is about 5.8 to 6.3 mm.[33]

The Paradise Renal Denervation System (Recor Medical) is a catheter-based system that delivers ultrasound energy circumferentially to thermally ablate the renal sympathetic nerves. The catheter has a low-pressure, water-filled cooling balloon to protect the renal arterial wall from thermal damage.[34] A minimum of 2 sonication sessions of 7 seconds each are delivered in the main renal artery, separated longitudinally by 5 mm. Accessory renal arteries also recommended for treatment if the diameter ranges from 3 mm to 8 mm. Penetration depth is about 6 mm.

The Peregrine System (Ablative Solutions) is the only catheter system that facilitates injection of dehydrated alcohol as a neurolytic agent into the renal periarterial space. This catheter has 3 co-planar micro-needles separated by 120 degrees. Alcohol is injected via these needles into the periarterial space to ablate the nerve fibers.[35] Each treatment involves the manual administration of 0.6 ml alcohol over 2 minutes, and alcohol injection is performed at the midpoint of the main renal artery. Penetration depth is approximately 3.5 mm.

Limitations and Future Developments

No objective metric exists to accurately indicate the degree or extent of actual nerve destruction. To overcome this issue, several catheter systems have been developed to indirectly detect complete denervation by measuring the pressure response to radiofrequency stimulation of the renal artery. This dynamic and transient potentiation of arterial pressure is presumably mediated by stimulation of the renal afferent nerves.[36,37] For example, the SyMapCath (Symap Medical) radiofrequency denervation system allows for radiofrequency renal artery stimulation prior to ablation in order to discriminate so called "hot" and "cold spots" associated with an increase, or lack of increase, in SBP during renal artery stimulation, respectively. These hot spots were ablated by the same catheter. In the SMART trial, the antihypertensive drug burden index was significantly lower in the RDN group, but there was no statistically significant difference in BP reduction compared to the sham procedure.[38]

Prior human studies have shown that renal artery stimulation before RDN typically resulted in an SBP increase of 24-50 mmHg. After successful RDN, this response was attenuated, with SBP increases typically reduced to 9-13 mmHg.[21,39] A research group in Taiwan has first shown that a maximal systolic BP recording of less than 20 mmHg following renal artery electrical stimulation after RDN was predictive of ambulatory and office BP reductions post-RDN.[21] The investigators emphasized that, to effectively implement renal artery stimulation as an intraprocedural feedback mechanism, it is crucial to standardize the stimulation protocol. Further, appropriate conscious anesthesia (typically intravenous propofol) is essential to mitigate the effects of pain-induced systemic vasovagal responses. The ongoing INFORM trial (INtraprocedural Feedback-Optimized Renal denervation based on Measurements obtained through renal artery stimulation, NCT06676553) as well as the InSight trial (NCT07013929) will each respectively evaluate whether renal artery electrical stimulation-directed RDN could further

improve clinical BP lowering responses.

Finally, currently available RDN systems are based on the trans-femoral arterial access approach. Considering the angle between the renal artery and the abdominal aorta, the trans-radial approach might be preferred for engaging the guiding catheter and maintaining the support of the guiding catheter in patients with sharp angle and/or tortuous infrarenal abdominal aorta.[40,41] Additionally, the trans-radial approach is associated with fewer bleeding complications. The Symplicity Spyral catheter can be used via the radial approach, provided that the length of the catheter is sufficient for the patient.[42]

References

1. Mahfoud F, Mancia G, Schmieder RE, Ruilope L, Narkiewicz K, Schlaich M, Williams B, Ribichini F, Weil J, Kao H-L, et al. Cardiovascular Risk Reduction After Renal Denervation According to Time in Therapeutic Systolic Blood Pressure Range. *J Am Coll Cardiol*. 2022;80:1871–1880.
2. Schmieder RE, Mahfoud F, Mancia G, Narkiewicz K, Ruilope L, Hutton DW, Cao KN, Hettrick DA, Fahy M, Schlaich MP, et al. Clinical event reductions in high-risk patients after renal denervation projected from the global SYMPLICITY registry. *Eur Heart J - Qual Care Clin Outcomes*. 2022;qcac056.
3. Katsurada K, Kario K. Effects of renal denervation on the incidence and severity of cardiovascular diseases. *Hypertens Res*. 2024;47:2700–2710.
4. Xie L, Li Y, Luo S, Huang B. Impact of renal denervation on cardiac remodeling in resistant hypertension: A meta-analysis. *Clin Cardiol*. 2024;47:e24222.
5. Bazoukis G, Thomopoulos C, Tse G, Vassiliou VS, Liu T, Dimitriadis K, Tatakis F, Konstantinou K, Doumas M, Tsioufis K. Impact of renal sympathetic denervation on cardiac magnetic resonance-derived cardiac indices in hypertensive patients – A meta-analysis. *J Cardiol*. 2021;78:314–321.
6. Wang S, Yang S, Zhao X, Shi J. Effects of Renal Denervation on Cardiac Structural and Functional Abnormalities in Patients with Resistant Hypertension or Diastolic Dysfunction. *Sci Rep*. 2018;8:1172.

7. Kordalis A, Tsiachris D, Pietri P, Tsioufis C, Stefanadis C. Regression of organ damage following renal denervation in resistant hypertension: a meta-analysis. *Journal of Hypertension*. 2018;36:1614–1621.

8. Li M, Ma W, Fan F, Yi T, Qiu L, Wang Z, Weng H, Zhang Y, Li J, Huo Y. Renal denervation in management of heart failure with reduced ejection fraction: A systematic review and meta-analysis. *J Cardiol*. 2023;81:513–521.

9. Schirmer SH, Sayed MMYA, Reil J-C, Ukena C, Linz D, Kindermann M, Laufs U, Mahfoud F, Böhm M. Improvements in Left Ventricular Hypertrophy and Diastolic Function Following Renal Denervation. *J Am Coll Cardiol*. 2014;63:1916–1923.

10. Mahfoud F, Urban D, Teller D, Linz D, Stawowy P, Hassel J-H, Fries P, Dreysse S, Wellnhofer E, Schneider G, et al. Effect of renal denervation on left ventricular mass and function in patients with resistant hypertension: data from a multi-centre cardiovascular magnetic resonance imaging trial. *Eur Heart J*. 2014;35:2224–2231b.

11. De Sousa Almeida M, De Araújo Gonçalves P, Branco P, Mesquita J, Carvalho MS, Dores H, Silva Sousa H, Gaspar A, Horta E, Aleixo A, et al. Impact of Renal Sympathetic Denervation on Left Ventricular Structure and Function at 1-Year Follow-Up. *PLoS ONE*. 2016;11:e0149855.

12. Li L, Xiong Y, Hu Z, Yao Y. Effect of Renal Denervation for the Management of Heart Rate in Patients With Hypertension: A Systematic Review and Meta-Analysis. *Front. Cardiovasc. Med.* 2022;8:810321.

13. Zeijen VJM, Theuns DA, Feyz L, Saville KA, Bhagwandien R, Kardys I, Van Mieghem NM, Daemen J. Long-term safety and efficacy of renal sympathetic denervation in atrial fibrillation: 3-year results of the AFFORD study. *Clin Res Cardiol*. 2023;112:1766–1777.

14. Chinitz L, Böhm M, Evonich R, Saba S, Sangriogoli R, Augostini R, O'Neill PG, Fellows C, Kim M-Y, Hettrick DA, et al. Long-Term Changes in Atrial Arrhythmia Burden After Renal Denervation Combined With Pulmonary Vein Isolation. *JACC: Clinl Electrophysio*. 2024;10:2062–2073.

15. Nawar K, Mohammad A, Johns EJ, Abdulla MH. Renal denervation for atrial fibrillation: a comprehensive updated systematic review and meta-analysis. *J Hum Hypertens*. 2023;37:89–90.

16. Hawson J, Harmer JA, Cowan M, Virk S, Campbell T, Bennett RG, Anderson RD, Kalman J, Lee G, Kumar S. Renal Denervation for the Management of Refractory Ventricular Arrhythmias. *JACC: Clin Electrophysio*. 2021;7:100–108.

17. Sanders MF, Reitsma JB, Morpey M, Gremmels H, Bots ML, Pisano A, Bolignano D, Zoccali C, Blankestijn PJ. Renal safety of catheter-based renal denervation: systematic review and meta-analysis. *Nephrol Dial Transplant*. 2017;32:1440–1447.

18. Mohammad AA, Nawar K, Binks O, Abdulla MH. Effects of renal denervation on kidney function in patients with chronic kidney disease: a systematic review and meta-analysis. *J Hum Hypertens*. 2024;38:29–44.

19. Xia M, Liu T, Chen D, Huang Y. Efficacy and safety of renal denervation for hypertension in patients with chronic kidney disease: a meta-analysis. *Inter J Hyperthermia*. 2021;38:732–742.

20. Kandzari DE, Bhatt DL, Brar S, Devireddy CM, Esler M, Fahy M, Flack JM, Katzen BT, Lea J, Lee DP, et al. Predictors of blood pressure response in the SYMPLICITY HTN-3 trial. *Eur Heart J*. 2015;36:219–227.

21. Mahfoud F, Cremers B, Janker J, Link B, Vonend O, Ukena C, Linz D, Schmieder R, Rump LC, Kindermann I, et al. Renal Hemodynamics and Renal Function After Catheter-Based Renal Sympathetic Denervation in Patients With Resistant Hypertension. *Hypertension*. 2012;60:419–424.

22. Doltra A, Hartmann A, Stawowy P, Goubergrits L, Kuehne T, Wellnhofer E, Gebker R, Schneeweis C, Schnackenburg B, Esler M, et al. Effects of Renal Denervation on Renal Artery Function in Humans: Preliminary Study. *PLoS ONE*. 2016;11:e0150662.

23. Yamazaki D, Konishi Y, Kitada K. Effects of renal denervation on the kidney: albuminuria, proteinuria, and renal function. *Hypertens Res*. 2024;47:2659–2664.

24. Gangemi C, Gambaro A, Ortalda V, Tavella D, Caletti C, Fezzi S, Bianco B, De Tomi E, Gambaro G, Ribichini FL, et al. Renal denervation in dialysis patients: long-term outcomes in a real-world setting. *Clin Kidney J*. 2025;18:sfaf149.

25. Zhang Z, Liu K, Xiao S, Chen X. Effects of catheter-based renal denervation on glycemic control and lipid levels: a systematic review and meta-analysis. *Acta Diabetol*. 2021;58:603–614.

26. Czerwieńska B, Lelek M, Gojowy D, Surma S, Mizia-Stec K, Więcek A, Adamczak M. Effect of Renal Denervation on the Plasma Adiponectin Concentration in Patients with Resistant Hypertension. *JCM*. 2023;12:2114.

27. Miroslawska AK, Gjessing PF, Solbu MD, Norvik JV, Fuskevåg OM, Hanssen TA, Steigen TK. Metabolic effects two years after renal denervation in insulin resistant hypertensive patients. The Re-Shape CV-risk study. *Clinical Nutrition*. 2021;40:1503–1509.

28. Eikelis N, Hering D, Marusic P, Duval J, Hammond LJ, Walton AS, Lambert EA, Esler MD, Lambert GW, Schlaich MP. The Effect of Renal Denervation on Plasma Adipokine Profile in Patients with Treatment Resistant Hypertension. *Front. Physiol.* 2017;8:369.

29. Kandzari DE, Townsend RR, Kario K, Mahfoud F, Weber MA, Schmieder RE, Pocock S, Tsioufis K, Konstantinidis D, Choi J, et al. Safety and Efficacy of Renal Denervation in Patients Taking Antihypertensive Medications. *J Am Coll Cardiol*. 2023;82:1809–1823.

30. Kandzari DE, Weber MA, Pathak A, Zidar JP, Saxena M, David SW, Schmieder RE, Janas AJ, Langer C, Persu A, et al. Effect of Alcohol-Mediated Renal Denervation on Blood Pressure in the Presence of Antihypertensive Medications: Primary Results From the TARGET BP I Randomized Clinical Trial. *Circulation*. 2024;149:1875–1884.

31. Schlaich MP, Sobotka PA, Krum H, Lambert E, Esler MD. Renal Sympathetic-Nerve Ablation for Uncontrolled Hypertension. *N Engl J Med*. 2009;361:932–934.

32. Mahfoud F, Townsend RR, Kandzari DE, Kario K, Schmieder RE, Tsioufis K, Pocock S, David S, Patel K, Rao A, et al. Changes in Plasma Renin Activity After Renal Artery Sympathetic Denervation. *J Am Coll Cardiol*. 2021;77:2909–2919.

33. Böhm M, Tsioufis K, Kandzari DE, Kario K, Weber MA, Schmieder RE, Townsend RR, Kulenthiran S, Ukena C, Pocock S, et al. Effect of Heart Rate on the Outcome of Renal Denervation in Patients With Uncontrolled Hypertension. *J Am Coll Cardiol*. 2021;78:1028–1038.

34. Zuern CS, Eick C, Rizas KD, Bauer S, Langer H, Gawaz M, Bauer A. Impaired Cardiac Baroreflex Sensitivity Predicts Response to Renal Sympathetic Denervation in Patients With Resistant Hypertension. *J Am Coll Cardiol*. 2013;62:2124–2130.

35. Hoogerwaard AF, De Jong MR, Adiyaman A, Smit JJJ, Delnoy PPHM, Heeg J-E, Van Hasselt BAAM, Ramdat Misier AR, Rienstra M, Van Gelder IC, et al. Renal sympathetic denervation induces changes in heart rate variability and is associated with a lower sympathetic tone. *Clin Res Cardiol*. 2019;108:22–30.

36. Fengler K, Rommel K, Hoellriegel R, Blazek S, Besler C, Desch S, Schuler G, Linke A, Lurz P. Pulse Wave Velocity Predicts Response to Renal Denervation in Isolated Systolic Hypertension. *JAHA*. 2017;6:e005879.

37. Fengler K, Rommel K-P, Kriese W, Kresoja K-P, Blazek S, Obradovic D, Feistritzer H-J, Lücke C, Gutberlet M, Desch S, et al. Assessment of arterial stiffness to predict blood pressure response to renal sympathetic denervation. *EuroIntervention*. 2022;18:e686–e694.

38. Wang J, Yin Y, Lu C, LuZ, Hu J, Wang Y, Ge J, Jiang H, Yao C, Yan X, et al. Efficacy and safety of sympathetic mapping and ablation of renal nerves for the treatment of

hypertension (SMART): 6-month follow-up of a randomised, controlled trial. *eClinicalMedicine*. 2024;72:102626.

39. Okon T, Röhnert K, Stiermaier T, Rommel K-P, Müller U, Fengler K, Schuler G, Desch S, Lurz P. Invasive aortic pulse wave velocity as a marker for arterial stiffness predicts outcome of renal sympathetic denervation. *EuroIntervention*. 2016;12:e684–e692.

40. Sata Y, Hering D, Head GA, Walton AS, Peter K, Marusic P, Duval J, Lee R, Hammond LJ, Lambert EA, et al. Ambulatory arterial stiffness index as a predictor of blood pressure response to renal denervation*. *J Hypertens*. 2018;36:1414–1422.

41. Weber T, Wassertheurer S, Mayer CC, Hametner B, Danninger K, Townsend RR, Mahfoud F, Kario K, Fahy M, DeBruin V, et al. Twenty-Four-Hour Pulsatile Hemodynamics Predict Brachial Blood Pressure Response to Renal Denervation in the SPYRAL HTN-OFF MED Trial. *Hypertension*. 2022;79:1506–1514.

42. Steinmetz M, Nelles D, Weisser-Thomas J, Schaefer C, Nickenig G, Werner N. Flow-mediated dilation, nitroglycerin-mediated dilation and their ratio predict successful renal denervation in mild resistant hypertension. *Clin Res Cardiol*. 2018;107:611–615.

43. Mufarrih S, Qureshi N, Khan MS, Kazimuddin M, Secemsky E, Bloch MJ, Giri J, Cohen D, Swaminathan RV, Feldman DN, et al. Randomized trials of renal denervation for uncontrolled hypertension: an updated meta-analysis. *J Am Heart Assoc* 2024; 13(16):e034910. doi: 10.1161/JAHA.124.034910.

44. Ogoyama Y, Abe M, Okamura K, Tada K, Katsurada K, Shibata S, Kai H, Rakugi H, Node K, Yokoi H, et al. Effects of renal denervation on blood pressure in patients with hypertension: a latest systematic review and meta-analysis of randomized sham-controlled trials. *Hypertens Res* 2024:47:2745-59. doi.org/10.1038/s41440-024-01739-y.

45. Sharp ASP, Sanderson A, Hansell N, Reddish K, Miller P, Moss J, Schmieder RE, McCool R.. Renal denervation for uncontrolled hypertension: a systematic review and meta-analysis examining multiple subgroups. *J Hypertens* 2024;**42**:1133-44. doi: 10.1097/HJH.0000000000003727.

46. Vukadinovic D, Lauder L, Kandzari DE, Bhatt DL, Kirtane AJ, Edelman ER, Schmieder RE, Azizi M, Böhm M, Mahfoud F. Effects of Catheter-Based Renal Denervation in Hypertension: A Systematic Review and Meta-Analysis. *Circulation* 2024;**150**:1599-611. doi: 10.1161/CIRCULATIONAHA.124.069709.

47. Krum H, Schlaich MP, Sobotka PA, Böhm M, Mahfoud F, Rocha-Singh K, Katholi R, Esler MD. Percutaneous renal denervation in patients with treatment-resistant hypertension: final 3-year report of the Symplicity HTN-1 study. Lancet 2014;383(9917):622-9. DOI: 10.1016/S0140-6736(13)62192

48. Esler MD, Böhm M, Sievert H, Rump CL, Schmieder RE, Krum H, Mahfoud F, Schlaich M. Catheter-based renal denervation for treatment of patients with treatment-resistant hypertension: 36 month results from the SYMPLICITY HTN-2 randomized clinical trial. *Eur Heart J* 2014;35:1752-9. DOI:10.1093/eurheartj/ehu209

49. Bhatt DL, Vaduganathan M, Kandzari DE, Leon MB, Rocha-Singh K, Townsend RR, Katzen BT, Oparil S, Brar S, DeBruin V, et al. Long-term outcomes after catheter-based renal artery denervation for resistant hypertension: final follow-up of the randomised SYMPLICITY HTN-3 Trial. *Lancet* 2022;400:1405-16. doi: 10.1016/S0140-6736(22)01787-1.

50. Kario K, Yamamoto E, Tomita H, Okura T, Saito S, Ueno T, Yasuhara D, Shimada K, SYMPLICITY HTN-Japan investigators. Sufficient and Persistent Blood Pressure Reduction in the Final Long-Term Results From SYMPLICITY HTN-Japan - Safety and Efficacy of Renal Denervation at 3 Years. *Circ J* 2019;83:622-9. doi: 10.1253/circj.CJ-18-1018.

51. Mahfoud F, Kandzari DE, Kario K, Townsend RR, Weber MA, Schmieder RE, Tsioufis K, Pocock S, Dimitriadis K, Choi JW, et al. Long-term efficacy and safety of renal denervation in the presence of antihypertensive drugs (SPYRAL HTN-ON MED): a randomised, sham-controlled trial. *Lancet* 2022; **399**(10333): 1401-10. doi: 10.1016/S0140-6736(22)00455-X.

52. Rader F, Kirtane AJ, Wang Y, Daemen J, Lurz P, Sayer J, Saxena M, Levy T, Scicli AP, Thackeray L et al. Durability of blood pressure reduction after ultrasound renal denervation: three-year follow-up of the treatment arm of the randomised RADIANCE-HTN SOLO trial. EuroIntervention 2022;18:e677-85. DOI: 10.4244/EIJ-D-22-00305

53. Mahfoud F, Böhm M, Schmieder R, Narkiewicz K, Ewen S, Ruilope L, Schlaich M, Williams B, Fahy M, Mancia G. Effects of renal denervation on kidney function and long-term outcomes: 3-year follow-up from the Global SYMPLICITY Registry. *Eur Heart J* 2019;40(42):3474-82. DOI: 10.1093/eurheartj/ehz118

54. Kim BK, Kim HS, Park SJ, Park CG, Seung KB, Gwon H-C, Chai D-J, Ahn TH, Kim CJ, Kwon H M, et al. Long-term outcomes after renal denervation in an Asian population: results from the Global SYMPLICITY Registry in South Korea (GSR Korea). *Hypertens Res* 2021; **44**(9): 1099-104. doi: 10.1038/s41440-021-00683-5.

55. Lee CK, Wang TD, Lee YH, Fahy M, Lee C-H, Sung S-H, Kao H-L, Wu Y-W, Lin T-H. Efficacy and Safety of Renal Denervation for Patients with Uncontrolled Hypertension in Taiwan: 3-Year Results From the Global SYMPLICITY Registry-Taiwan (GSR-Taiwan). *Acta Cardiol Sin* 2019; **35**(6): 618-26. doi: 10.6515/ACS.201911_35(6).20190826A.

56. Zeijen VJM, Feyz L, Nannan Panday R, Versmissen J, Kardys I, Van Mieghem N, Daemen J. Long-term follow-up of patients undergoing renal sympathetic denervation. *Clin Res Cardiol* 2022;111(11):1256-68. doi: 10.1007/s00392-022-02056-5

57. Panchavinnin P, Wanthong S, Roubsanthisuk W, Tresukosol D, Buranakitjaroen P, Chotruangnapa C, Watanapa W, Pongakasira R, Wongpraparut N. Long-term outcome of renal nerve denervation (RDN) for resistant hypertension. *Hypertens Res* 2022;45:962-6. doi: 10.1038/s41440-022-00910-7.

58. Sesa-Ashton G, Nolde JM, Muente I, Carnagarin R, Lee R, Macefield VG, Dawood T, Sata Y, Lambert EA, Lambert GW, et al. Catheter-Based Renal Denervation: 9-Year Follow-Up Data on Safety and Blood Pressure Reduction in Patients With Resistant Hypertension. *Hypertension* 2023;80:811-9. doi: 10.1161/HYPERTENSIONAHA.122.20853.

59. Al Ghorani H, Kulenthiran S, Lauder L, Recktenwald MJM, Dederer J, Kunz M, Götzinger F, Ewen S, Ukena C, Böhm M, et al. Ultra-long-term efficacy and safety of catheter-based renal denervation in resistant hypertension: 10-year follow-up outcomes. *Clin Res Cardiol* 2024;113:1384-92. doi: 10.1007/s00392-024-02417-2.

60. Wang L, Li C, Li Z, Li Q, Liu C, Sun X, He Q, Xia D-S, Xia D, Lu C. Ten-year follow-up of very-high risk hypertensive patients undergoing renal sympathetic denervation. *J Hypertens* 2024;**42**:801-8. doi: 10.1097/HJH.0000000000003650.

61. Chinese Society of Cardiology CMA, Hypertension Committee of Cross-Straits Medicine Exchange A, Cardiovascular Disease P, Rehabilitation Committee CAoRM. Clinical practice guideline for the management of hypertension in China. *Chin Med J (Engl)* 2024;**137**:2907-52.

62. Umemura S, Arima H, Arima S, et al. The Japanese Society of Hypertension Guidelines for the Management of Hypertension (JSH 2019). *Hypertens Res* 2019;**42**:1235-481.

63. Kim HC, Ihm SH, Kim GH, et al. 2018 Korean Society of Hypertension guidelines for the management of hypertension: part I-epidemiology of hypertension. *Clin Hypertens* 2019;**25**:16.

64. Wang TD, Chiang CE, Chao TH, et al. 2022 Guidelines of the Taiwan Society of Cardiology and the Taiwan Hypertension Society for the Management of Hypertension. *Acta Cardiol Sin* 2022;**38**:225-325.

65. Ona DID, Jimeno CA, Jasul GV, Jr., et al. Executive summary of the 2020 clinical practice guidelines for the management of hypertension in the Philippines. *J Clin Hypertens (Greenwich)* 2021;**23**):1637-50.

66. Kario K, Kandzari DE, Mahfoud F, Weber MA, Schmieder RE, Tsioufis K, Liu M, Böhm M, Townsend RR. Renal Denervation Lowers Nighttime Blood Pressure in True Resistant Hypertension. *Hypertension*. 2024;81:e200-e202.

67. Kario K. Catheter-Based Renal Denervation Ready for the Management of Hypertension: Evidence, Challenges, and Perspectives. *J Am Heart Assoc*. 2024;13(16):e037099

68. Kario K, Tomitani N, Nishizawa M, Harada N, Kanegae H, Hoshide S. Concept, study design, and baseline blood pressure control status of the nationwide prospective HI–JAMP study using multisensor ABPM. Hypertens Res. 2023;46:357–367.

Table S1. Summary of random effect sizes for recent meta-analyses

**Lead author**	**Office SBP Mean effect [95% CI]**	**24-hr SBP Mean effect [95% CI]**
Mufarrih S[43]*	−6.39 [95% CI, −11.49, −1.30] mmHg	−2.23 [95% CI, −3.56, −0.90] mmHg
Ogoyama Y[44]	-4.95 [95% CI, -6.37, -3.54] mmHg	-2.81 [95% CI: -4.09, -1.53] mmHg
Sharp ASP[45]	-8.5 [95% CI, -13.5, -3.6] mmHg	-3.6 [95% CI, -5.2, -2.0] mmHg
Vukadinovic D[46]	-6.62 [95%CI, -9.66, -3.57] mmHg	-4.41 [95% CI: -6.18, -2.70] mmHg

*ON MED trials

All random effect values are sham-control adjusted.

Table S2. Long-term efficacy and safety of RDN including non-sham-controlled trials and registries

Trials	RDN energy	Study design	No. patients	Population	Primary outcome	Follow-up (months)	Efficacy	Safety
**Symplicity HTN-1**[47]	RFA	Prospective, open-label registry	88/153	Severe resistant HTN	OBP	36	Office SBP/DBP: -32/ -14 mmHg	1 new RAS
**Symplicity HTN-2**[48]	RFA	Multi-center randomized trial	70/106	Uncontrolled HTN (SBP>160, >3 meds)	OBP	36	Office SBP/DBP: -33/ -14 mmHg	1 hematoma 1 dissection
**Symplicity HTN-3**[49]	RFA	Multi-center, randomized single blinded, sham-controlled trial	315/535	Resistant hypertension on stable doses	OBP and 24-h ABPM	36	(RDN vs Sham) Office SBP: -26.4 vs -5.7 mmHg 24-h ABPM: -15.6 vs -0.3 mmHg	Similar composite safety endpoint*
**Symplicity HTN-J**[50*]	RFA	Multi-center randomized trial	41	Uncontrolled HTN (SBP>160, >3 meds)	OBP	36	Office SBP/DBP: -32.8/-15.8 mmHg	None

**SPYRAL ON-MED**[51]	RFA	Multi-center, randomized single blinded, sham-controlled	80	Uncontrolled HTN	24-h ABPM	36	Morning SBP: -11.0 mmHg Night SBP: -11.8 mmHg Day SBP: -5.8 mmHg	None
**RADIANCE SOLO**[52]	Ultrasound	Multi-center, randomized single blinded, sham-controlled	51/74	HTN ABPM 135-170/85-105	Office BP	36	-18 mmHg	None
**Global Symplicity Registry (GSR)**[53]	RFA	Prospective, open-label registry	1742/2237	Hypertensive patients received RDN	Office BP 24-h ABPM	36	Office SBP: -16.5 mmHg 24-h SBP: -8.0 mmHg	Renal re-intervention 0.6%
**GSR Korea**[54*]	RFA	Prospective, open-label registry	102	Uncontrolled HTN (SBP>160, >3 meds)	% of pts with >10 mmHg Office SBP reduction	36	89.7%	AE-rare

**GSR Taiwan**[55*]	RFA	Prospective, open-label registry	26	Resistant HTN	Office SBP	36 (for Flex) 24 (for Spyral)	-29.7 mmHg (for Flex) -42.4 mmHg (for Spyral)	None
**Erasmus Registry**[56]	RFA Ultrasound	Prospective, open-label registry	72	Hypertensive patients received RDN	24-h ABPM	48 (Median) 60 (longest)	24-h SBP: -20.9 mmHg 24-h DBP: -8.3 mmHg	4.2% peri-procedural complication None long-term
**Siriraj RDN**[57*]	RFA	Prospective, open-label registry	18	Refractory HTN/severe resistant HTN	% of pts with >10 mmHg OSBP reduction or reduced medication	54 (mean) 104 (longest)	After 3 yr 92-100%	1 patient resurgence BP required repeat RDN
**Alfred Hospital**[58]	RFA	Various RDN trial	66	Resistant HTN	24 ABPM	108	240h SBP: -12.1 mmHg 24-h DBP: -8.8 mmHg	4 MACEs
**Saarland University Hospital**[59]	RFA	Single-center, real-world observation	39	Resistant HTN	Office BP 24 ABPM	120	Office SBP: -16 mmHg 24-h SBP: -14 mmHg	3 renal artery intervention cases

Tianjin First Central Hospital[60*]	RFA	Single-center, real-world observation	60	Resistant HTN	Office BP 24 ABPM	120 (mean) 144 (longest)	After 10 years, Office SBP: -35.9 mmHg 24-h SBP: -14.3 mmHg	11 non-fatal MACE, 6 dead after 10 years

*All participants from countries/regions in Asia

Number x/xx= number of patients at follow-up period/number of patients at enrollment

Table S3. Summary of renal denervation methodologies.

	Radiofrequency	Ultrasound	Alcohol
Approach	Femoral (Radial possible)	Femoral	Femoral
French size	6Fr	7Fr	7Fr
Treatable renal artery diameter (mm)	3-8	3-8	3-7
Ablation point	Main and distal branch RA	Main RA	Mid segment of main RA
Each ablation/sonication time or injected volume	1 minute	7 seconds<br>maximum 4 sonication in main RA<br>1 sonication in accessory RA	0.6 ml dehydrated alcohol<br>maximum 2.4 ml per patient<br>1 injection in each main and accessory RA
Penetration depth (mm)	5.8-6.3 mm	6 mm	3.5 mm
FDA approval	November 2023	November 2023	-
Pivotal study	SPYRAL-HTN OFF MED	RADIANCE II/HT-SOLO/ HT-TRIO	TARGET BPI
Total ablation, sonication, or injected number	47.4+/-16.5	5.6+/-1.1	2.2

Accessory renal artery treated	26.0%	19.1%	21.6%
Procedural time (minute)	91.3+/-31.2	78.1+/-25.8	55.7+/-27.0
Catheter time (minute)	54.5+/-19.2	35.3+/-20.1	-
Contrast Volume (ml)	204.2+/-81.4	146.7+/-71.9	95.7+/-47.4
Procedure success rate	99.5%	97.6%	92.7%

RA: Renal Artery, FDA: Food and Drug Administration

Table S4. Recommendations of the recent Asian hypertension guidelines for the use of renal denervation in the treatment of hypertension

Guideline	Publication year	Recommendations
China[61]	2024	RDN can be considered for hypertensive patients with drug-resistant hypertension or poor adherence to medication, after excluding secondary hypertension (IIb, B).<br><br>RDN should be performed in departments with extensive experience in the diagnosis and treatment of hypertension, and with the capacity to differentiate the causes of secondary hypertension (I, C).
Japan[62]	2019	RDN may be useful as a non-pharmaceutical procedure for hypertension treatment in patients with mild hypertension in addition to those with resistant hypertension.
Korea[63]	2018	Carotid baroreceptor stimulation or renal denervation may be tried in patients with true resistant HTN since the risk of procedure-related complication is low. However, there are non-responders and a lack of evidence of long-term effects, therefore it is not currently recommended.
Taiwan[64]	2022	Renal denervation can be considered as a BP-lowering strategy in hypertensive patients with high CV risk, such as resistant or masked uncontrolled hypertension, established ASCVD, intolerant or nonadherent to antihypertensive drugs, or features indicative of neurogenic hypertension after careful clinical and imaging evaluation (COR IIa, LOE B).
The Philippines[65]	2020	None.

Guidelines are listed in the alphabetical order of the name of countries/regions.

Table S5. Future Goals of ARDeC (Asia Renal Denervation Consortium)

Area	Goals
Clinical Research	Conduct long-term cardiovascular outcome studies in Asian populations
	Investigate RDN efficacy in Asian-specific hypertension phenotypes (salt-sensitive, nocturnal, morning hypertension)
	Evaluate predictors of treatment response in Asian populations
Standardization	Establish standardized patient selection protocols across Asia
	Develop unified procedural techniques and quality metrics
	Create standardized follow-up protocols
Healthcare Integration	Develop cost-effectiveness data specific to Asian healthcare systems
	Create clear guidelines for integrating RDN into existing treatment pathways
	Work towards reimbursement approval in various Asian healthcare systems
Data Collection	Build comprehensive Asian RDN registry
	Continued collection of real-world evidence on safety and efficacy
	Monitor long-term outcomes in Asian populations
Education & Collaboration	Foster expertise sharing between Asian centers
	Standardize training requirements for operators
	Promote regional collaboration in research and practice

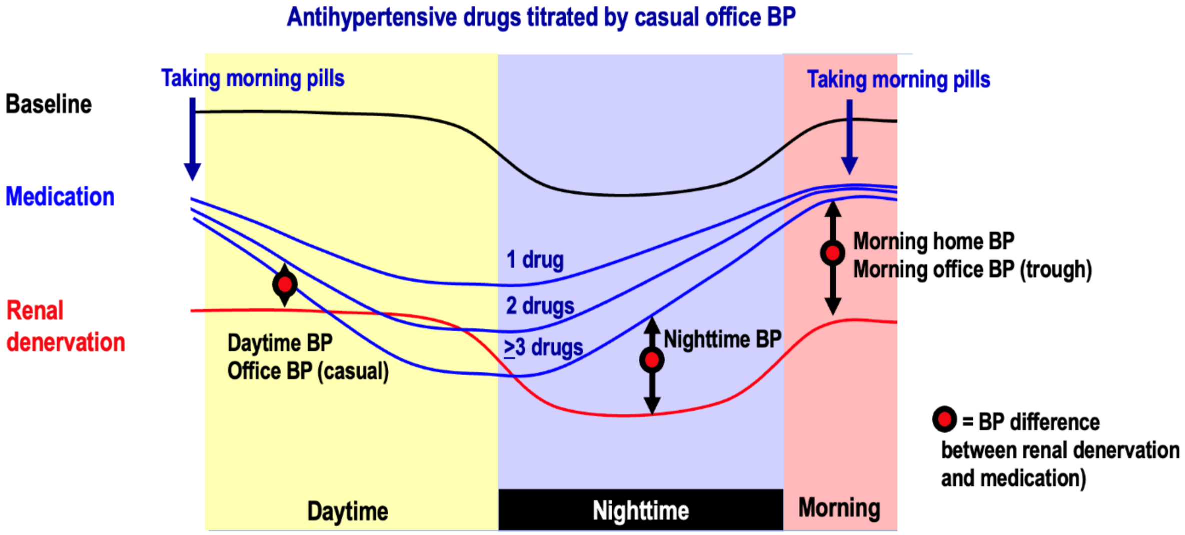

Figure S1. Blood pressure (BP)-lowering effects of antihypertensive medications compared with the "always-on" 24-hour BP-lowering effects of renal denervation (including nighttime and morning BP values).[66] Reprint from Kario K. 2024.[67]

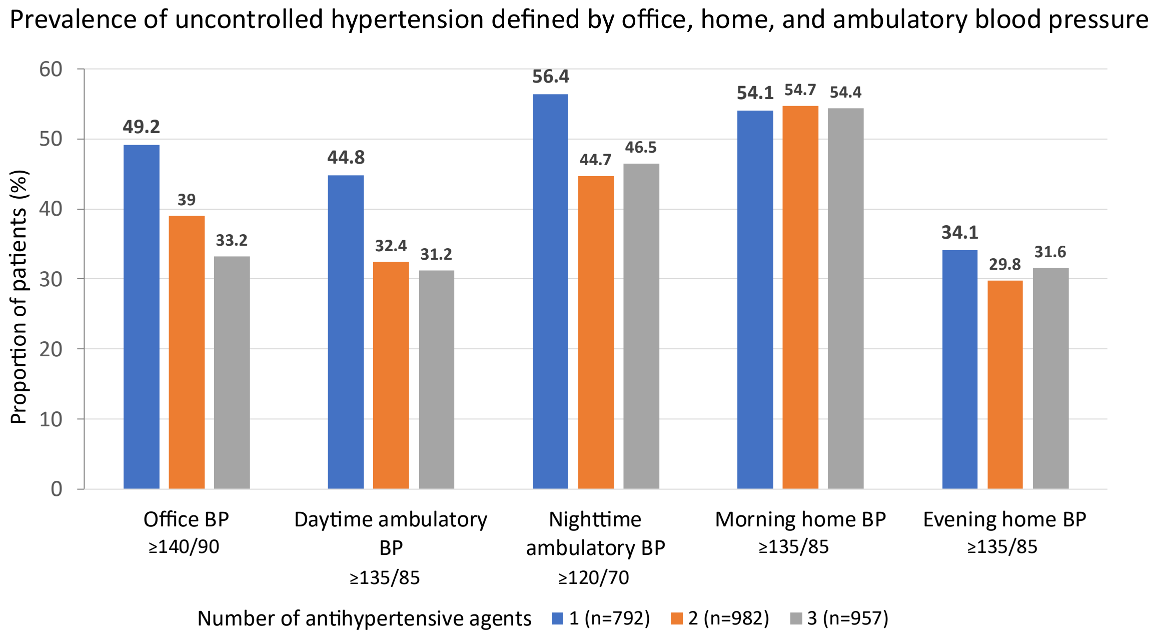

Figure S2. Prevalence of uncontrolled hypertension defined by office, home, and ambulatory blood pressure (BP) measurements determined using an 'all-in-one' monitoring device (data from the Home-Activity Information and Communication Technology [ICT]-based Japan Ambulatory Blood Pressure Monitoring Prospective [HI–JAMP]) study[68]; Reprint from Kario K. 2024.[67])
